# Prevalence of Stroke and Hypoperfusion in Patients With Isolated Vertigo and Vascular Risk Factors

**DOI:** 10.3389/fneur.2018.00974

**Published:** 2018-11-15

**Authors:** Dao Pei Zhang, Hao Ran Li, Qian Kun Ma, Suo Yin, Yan Fang Peng, Huai Liang Zhang, Min Zhao, Shu Ling Zhang

**Affiliations:** ^1^Department of Neurology, The First Affiliated Hospital of Henan University of Chinese Medicine, Zhengzhou, China; ^2^Department of Neurology, People's Hospital of Zhengzhou Affiliated to Southern Medical University, Zhengzhou, China; ^3^Department of Neurology, People's Hospital Affiliated to Zhengzhou University, Zhengzhou, China; ^4^Department of Image, People's Hospital of Zhengzhou Affiliated to Southern Medical University, Zhengzhou, China

**Keywords:** vertigo, stroke, hypoperfusion, vertebral artery, basilar artery, vessel curvature

## Abstract

**Background and Purpose:** The aim of this study was to determine the prevalence and associated factors of stroke and hypoperfusion among patients with isolated vertigo and vascular risk factors.

**Methods:** We studied 157 patients with isolated vertigo who had undergone multimodal magnetic resonance imaging. Magnetic resonance angiography (MRA) was used to measure the diameters of vertebrobasilar arteries and to evaluate morphologic changes to vessels. Measurements obtained included length of the basilar artery and curvature index for the vertebral artery (VA). Perfusion-weighted imaging (PWI) was performed to determine relative cerebral blood flow, relative cerebral blood volume (rCBV), time to peak (TTP), and mean transit time for two mirror regions of interest (ROIs) in each map. Regional hypoperfusion of the cerebellum was considered significant when TTP and mean transit time (MTT) were present in ≥2 adjacent slices.

**Results:** The prevalence of stroke in patients with isolated vertigo and vascular risk factors was 24.8% (*n* = 39). Visual assessment revealed cerebellar hypoperfusion in 57.6% (68/118) of non-stroke patients. Multivariate logistic regression indicated that diabetes mellitus (*P* = 0.049, OR = 2.758), VA stenosis or hypoplasia (*P* = 0.023, OR = 3.486), and relative TTP of cerebellum (*P* = 0.002, OR = 3.197) were independent risk factors for stroke and LVA curvature index (*P* = 0.026, OR = 2.049), VA stenosis and hypoplasia (*P* = 0.009, OR = 2.977) were independent risk factors for hypoperfusion.

**Conclusions:** The prevalence of stroke and hypoperfusion is higher in patients with isolated vertigo and vascular risk factors, compared with matched controls. Potential risk factors include diabetes mellitus, VA stenosis or hypoplasia, and enlarged VA curvature index.

## Introduction

Patients with acute isolated vertigo have vertigo without focal neurological symptoms (unsteady gait and nystagmus are allowed) (1, 2) involving or preceded by stroke/transient ischemic attack (TIA) (3). Diagnosis of vertigo in patients presenting with posterior circulation stroke/TIA has recently increased markedly (4, 5). A population-based registry showed that 90% of isolated posterior circulation TIAs (half of which were associated with isolated vertigo symptoms), were not recognized when the patient first presented to a health-care practitioner (6). One retrospective recently reported that ~20% of patients who have had a brainstem stroke report having also had isolated attacks of vertigo (7). Regretfully, the diagnosis of stroke in patients with acute isolated vertigo remains a challenge when the diagnosis is based on bedside examination findings and routine MRIs (3). Application of HINTS (head impulse, nystagmus patterns, test of skew) has greatly enhanced the diagnosis of stroke in acute isolated vertigo. However, HINTS could not be applied to the majority of patients with isolated vertigo because the vestibular symptoms or signs had already resolved in about 70% of patients (1). Although diffusion-weighted imaging magnetic resonance (DWI-MR) remains the standard modality with which to confirm acute infarction. However, when performed within 24–48 h of vertigo onset, images obtained with DWI-MR are falsely negative for ~20% of patients with clinically verified vertebrobasilar stroke (1, 8).

An important recent study that included initial and follow-up images indicated that 12% of patients showed unilateral cerebellar hypoperfusion on perfusion-weighted imaging (PWI) without an infarction on DWI (1). The results obtained with CT perfusion showed that unilateral hypoperfusion of the cerebellum and brainstem heralds the occurrence of isolated vertigo (3). Another study found that disequilibrium in the relative mean transit time (rMTT) of the bilateral cerebellum (as demonstrated with PWI) that hypoperfusion could trigger recurrent isolated vertigo (9). The combination of perfusion images analysis and clinical assessment may help to distinguish an attack of vertigo from a subtle stroke/TIA, it remains difficult to identify useful predictors of stroke in patients with isolated vertigo (10, 11).

The present study aims to characterize the prevalence of stroke and hypoperfusion and to analyze clinical findings and radiographic images to facilitate early detection of infarction or hypoperfusion in patients with isolated vertigo and vascular risk factors. To achieve this, we sought to identify traditional cerebrovascular risk factors based on clinical presentation, results of bedside examinations, and morphological changes in vertebrobasilar arteries as determined by magnetic resonance angiography (MRA). Using PWI, we characterized perfusion in the internal inferior cerebellum, dorsolateral medulla, occipital and temporal lobes, pons at the level of the vestibular nucleus, and thalamus in the plane of the internal capsule.

## Methods

### Ethical considerations

This prospective, single-center, observational study was approved by the Institutional Ethics Committee of People's Hospital of Zhengzhou, China. Each participant in the study provided informed consent.

### Experimental design and patient population

This was a prospective study that included four groups of patients with suspected vascular isolated vertigo. The stroke group included patients suffering from isolated vertigo caused by vascular disease as demonstrated by neuroimaging (typically DWI-MR). The non-stroke group comprised participants whose isolated vertigo was clinically suspected to have a vascular etiology, despite negative findings on DWI-MR. Patients in the non-stroke group were further classified, based on PWI findings, as hypoperfusion or non-hypoperfusion.

A total of 279 patients seen at the Department of Neurology at Zhengzhou People's Hospital were enrolled during the period from November 2014 to February 2017. Inclusion criteria were as follows: (1) acute onset of vertigo suspected to result from vascular ischemia; (2) no focal neurological signs or symptoms except for unsteady gait, nystagmus, intolerance of head motion, autonomic nervous symptoms or tinnitus; (3) at least one cerebrovascular risk factor such as hypertension, diabetes or elevated blood glucose, dyslipidemia disorder, coronary atherosclerotic heart disease, history of stroke, hyperhomocysteinemia, smoking, and alcoholism; (4) age >18 years; (5) positional vertigo considered to be independent of benign paroxysmal positional vertigo, Meniere disease, and vestibular neuritis (based on clinical symptoms and signs, if the results of vestibular function tests were negative). Patients were excluded if they had migraine (*n* = 12), sever emotional disease (*n* = 1), cognitive disorder (*n* = 1), compression of the eighth cranial nerve (*n* = 3), trauma(*n* = 1), hemorrhage (*n* = 5), taken drugs that might induce vertigo within the past 2 weeks (*n* = 4), subclavian steal syndrome (*n* = 2), not undergo DWI-MR (*n* = 4), contrast-enhanced MRA (*n* = 3), Meniere's disease (*n* = 8), vestibular neuritis (*n* = 19), benign paroxysmal positional vertigo (*n* = 42), and other otorhinolaryngology disease (*n* = 1). PWI was performed to evaluate the remaining 162 patients. However, for 5 patients, the quality of PWI images was poor; these patients were excluded. In the last, there remained 157 patients with isolated vertigo with at least one vascular risk factor and with intact data.

For the purpose of this study, the following factors were evaluated: history of hypertension [previous diagnosis of arterial hypertension: systolic blood pressure > 140 mmHg and/or diastolic >90 mmHg, or use of antihypertensive agents (past or present)], diabetes mellitus (previous diagnosis of diabetes or past or present use of antidiabetic agents), hyperlipidemia (cholesterol >5.17 mmol/L and/or triglycerides >1.71 mmol/L), hyperhomocysteinemia (>15.0 μmol/L), consumption of alcohol at least once a week (1 standard alcohol consumption is equivalent to 120 mL of wine, 360 mL of beer, or 45 mL of distilled spirits), smoking (continuous or cumulative history of smoking >6 mo and ≥1 cigarette per day), history of coronary artery disease (CAD), and presence of cerebrovascular stenosis (>50% stenosis of ≥1 intracranial artery, according to MRA). All patients with cerebrovascular risk factors had been previously diagnosed as such and/or were already taking medications for these conditions.

## MR examination protocol and image processing

MRI was performed in a 3.0 T gradient-echo (GE) Signa HDX (Fairfield, USA). The acquisition sequence included T1- and T2-weighted imaging, fluid attenuated inversion recovery (FLAIR) imaging and DWI and apparent on diffusion coefficient (ADC). DWI was acquired using spin-echo planar imaging sequence with TR 50000, TE 77.6, field of view 220 × 220 mm and slice thickness of 5.0 mm. At the same time, cervical CE-MRA was performed with injection of gadopentetate dimeglumine and three-dimensional time-of-flight (3D-TOF) MRA was also performed in the HDX with a repetition time of 24 ms, TR of 4.8, echo time of 6 ms, TE of 1.6/Fr, field of view of 320 × 320 mm and section thickness of 0.8–1.6 mm. A definite infarction was made when the lesion with high signal on DWI and low signal on ADC.

The means of three dimensional maximum intensity projection (3D-MIP) was used in the imaging reconstruction and data acquisition and we done the work on a post-processing GE machine (Siemens, Inc., Munich, Germany) with the AW Volume share 5 software (version 9.4.05). Measurement information was collected by two experienced observers in order to reduce bias. The diameter of all four segments of both sides of vertebral artery (V1–V4) and the diameter of basilar artery were measured in the reformed window with the same setting. Vertebral artery segments were classified as follows: V1, from origin to the transverse foramina of C5 or C6 vertebrae, V2, from the transverse foramina of C5 or C6 to the transverse foramina to C2, V3, from the C2 transverse foramina to dura, V4, from dura to the confluence of 2 vertebral arteries to form the basilar artery (12, 13).

**Figure 1:**
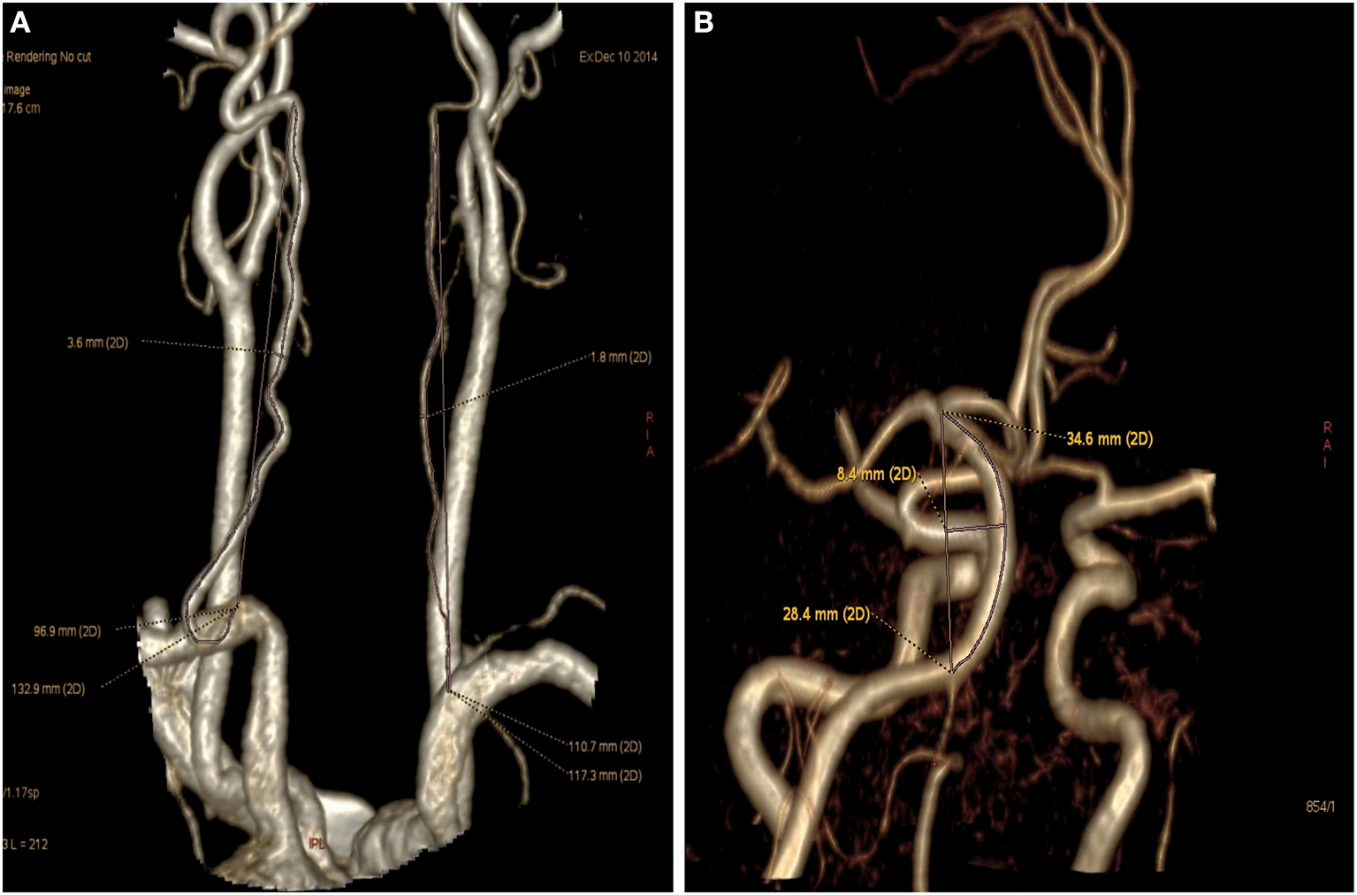
Vertebral artery and basilar artery curvature measured. **(A)** Left vertebral artery (V1-V2) actual length was 132.9 mm and straight length was 96.9 mm (Curvature index of VA = 132.9 – 96.9/96.9 × 100% = 37.2%), right hypoplastic vertebral artery actual length was 117.3 mm and straight length was 110.7 mm. **(B)** Basilar artery actual length was 34.6 mm, basilar artery straight length was 28.4 mm, basilar bending length was 8.4 mm.

Diameter of the basilar artery was measured at 3 locations: the point of junction with the vertebral artery, the top point, and the middle point. The average of these 3 values was taken as mean diameter of the basilar artery. In addition, the morphological changes of vertebrobasilar artery system measured also included actual and straight vertebral artery length (V1–V2; Figure 1A), basilar artery actual and straight length, basilar artery bending length (Figure 1B), and angle degree of vertebral artery (14). Basilar artery bending length refers to the distance from the basilar artery bending point to the basilar standard line. Vertebral artery hypoplasia (VAH) was defined as a V4 diameter ≤ 2 mm, slim or absent, or an asymmetry ration for the two sides of vertebral arteries >1:1.7 (15). Ratio of BA length = Actual length of BA/straight length of BA. Curvature index of VA = (actual length—straight length)/(straight length) × 100% (16). Patients were also classified according to the existence vs. lack of a fetal posterior cerebral artery. In accordance with North American Symptomatic Carotid Endarterectomy Trial method, vertebral artery stenosis was calculated as: (normal diameter at the distal end—narrowest diameter)/normal diameter at the distal end × 100%.

### PWI examination and obtaining parameter values

**Figure 2:**
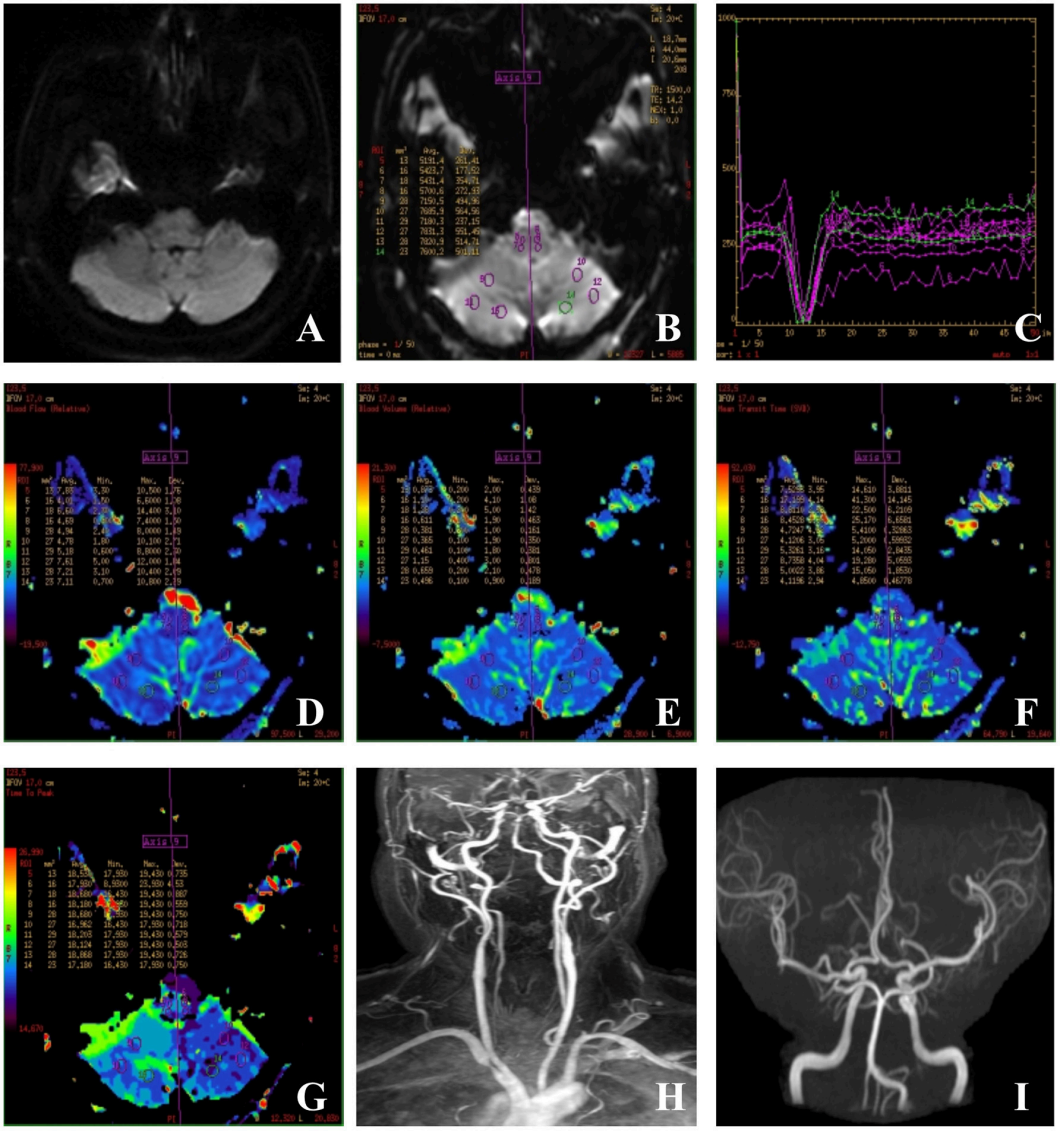
Images of a case of isolated vertigo suspected to be caused by hypodynamics of brain. **(A)** Sixty-one-year-old man had isolated vertigo on 4 April 2016 and performed with MRI, MRA, CEMRA, and PWI. **(A)** DWI-MRI was normal and showed no acute infarctions; **(B)** outlines of ROIs of bilateral cerebellum and medulla; **(C)** time-signal intensity curve; **(D,E)** cerebral blood flow (CBF) and cerebral blood volume: a similar color differences were not observed in the rCBF and rCBV panels. **(F)** Mean transit time (MTT): values of the right cerebellum varied from the left ones and are not obvious; **(G)** time to peak (TTP): TTP values obtained for the right cerebellum are longer than those obtained for the left cerebellum; **(H,I)** CEMRA and MRA showed the tortuous, dominant left vertebral artery, and severe stenosis at the beginning point.

PWI were obtained from dynamic susceptibility contrast-enhanced perfusion MR images using a gradient-echo echo planar image technique measuring the variation of signal intensity. The contrast agent used in our study was gadopentetate dimeglumine (Gd-DTPA), which can alter the local magnetic filed and therefore cause the decrease of signal intensity in the surrounding brain tissue. Because scan protocols and post-processing methods vary widely among manufacturers, the following scanning parameters were used: TR 1,500 ms, TE 15.2 ms, field of view 24 × 24 mm, section thickness of 5 mm with a 1.5 mm gap. During scanning, 0.2 mL/kg Gd-DTPA was injected at a rate of 4 mL/s; this was followed with an equivalent dose of saline. In the last, a total of 1,200 images were obtained from 24 slices and 50 repeated consecutive times and the original images were processed by the GE Brainstat software application (Functool 9.4.05). The AIF protocol was used to generate processed maps of relative cerebral blood flow (rCBF), relative cerebral blood volume (rCBV), mean transit time (MTT), and time to peak (TTP) (Figure 2). The AIF protocol is based on artery input functions and vein output functions; all measurements were performed by an experienced neuroradiologist. PWI was obtained within 24 h from the symptom onset in 82%, and within 48 h in 94%. The remaining 3 patients received PWI between 3 and 4 days from the symptom onset. The results of PWI were independently assessed by a neurologist (Y. S.) and neuroradiologist (M.Q.K.) who were blinded to all clinical information. They visually assessed the presence of asymmetrical hypoperfusion in the cerebellum using TTP and MTT maps (Figure 2). A regional hypoperfusion was considered significant when it was present in ≥2 adjacent slices. If there were any disagreements between the 2, we made a final decision based on consensus. They did not visually assess the medulla oblongata, pons and thalamus because of not easy to visually assess by PWI. All patients with cerebellar hypoperfusion on PWI had received follow-up PWI within 3 month after resolution of the symptoms. We considered cerebellar hypoperfusion as a cause of isolated vertigo in patients with vascular risk factors only when the symptoms of vertigo were not recurrent and the initial hypoperfusion was normalized on the follow-up images.

The regions of interest (ROIs) were located in the internal inferior cerebellum, dorsolateral medulla, occipital and temporal lobes, corona radiata, pons at the level of the vestibular nucleus, and thalamus in the plane of the internal capsule. Mirrored ROIs were automatically created on the same plane in the position of symmetry, after the axis of symmetry was created. Each ROI was outlined in the map of CBF firstly in reference to MTT to avoid the large vessels and then automatically copied onto the CBV, MTT, and CBV panels. Considering the physiological differences, the impact of hemodynamic constants and allowing convenient comparisons between individuals, the relative values of the two mirror ROI of each map were calculated including rrCBF, rrCBV, rMTT, and rTTP (= the absolute difference of parameter value between left to right ROI), which represent the absolute difference between the two symmetry ROI.

To assess the reliability of PWI, we also performed PWI in 32 patients with peripheral vestibulopathy. Patients were considered to have peripheral vestibulopathy if MRI and DWI scanning images were negative but tests of vestibular function were positive.

### Statistical analysis

Statistical analyses were performed using SPSS 19.0 software (IBM, Chicago, IL, USA). Normally distributed continuous variables were expressed as mean ± standard deviation and independent-samples *t*-test was used for two groups comparison, one-way analysis of variance (ANOVA) for three or more groups comparison. Whereas, non-normal distribution parametric were presented as median (range) if not passed the normal distribution test, and Mann-Whitney *U*-test were used for the comparison between the two groups, while to compare the differences among three groups or more, Kruskal-Wallis H test were used. Categorical variables were expressed as percentage and the chi square test was used in all the test of rate. According to the sample size and expected value, special chi square formula, the correction formula or the exact probability formula were used, respectively. In all of the tests, *P* < 0.05 were considered statistically significant. Variables with a *P* < 0.05 in the univariate analysis were selected and evaluated by multivariate logistic regression models with the forward stepwise selection method.

## Results

### General demographic findings

In total, 157 eligible patients with isolated vertigo, aged 28–90 years (median, 59.9 ± 12.6 years) were included in the study. Male patients accounted for 49.7% of the total. Among 157 patients, brain DWI-MR revealed recent ischemic stroke in 39 (24.8%) patients. In the remaining 118 patients without infarction lesions, PWI revealed cerebellar hypoperfusion by visually assessed in 68 patients (57.6%, 68/118). Ten of the patients also had a reduced CBF with normal CBV in the corresponding areas. Forty patients had a perfusion defect in the area of the medial posterior inferior cerebellar artery; the remaining 28 perfusion defects were found in the whole posterior inferior cerebellar artery. Among 68 patients, 28 (41.2%) had not recurrent vertigo symptoms and showed a perfusion defect in unilateral or bilateral cerebellum on TTP and MTT maps and were shown to normalize the initial cerebellar hypoperfusion without a complete infarction on the follow-up images. PWI revealed a perfusion defect in 2 of the 32 patients with peripheral vestibulopathy.

#### Analysis of infarcts

Of the 39 infarcted patients, infratentorial lesions, and supratentorial lesions were both found. The stroke lesions in our study involved were cerebellum (*n* = 25). Eighteen patients had infarctions restricted to the unilateral (*n* = 13) or bilateral (*n* = 5) cerebellum, whereas 7 had additional infarctions in the frontal (*n* = 1), thalamus (*n* = 3) or occipital lobe (*n* = 3), brainstem (3 pons, 2 medulla oblongata, 1 mesencephalon, 1 mesencephalon association with pons), pedunculus cerebellaris medius (*n* = 2), splenium of corpus callosum (*n* = 2), and temporoparietal (*n* = 3) area. Among 39 patients, 16 had acute transient vertigo, and 23 had continuous vertigo (>24 h). In patients with transient vertigo, infarcts usually occurred in the area of the posterior inferior cerebellar artery (cerebellum; *P* = 0.04); in patients with persistent vertigo, infarcts were typically found in the area of the basilar artery (brainstem; *P* = 0.01; Table 1).

**Table 1:** Analysis of infarct lesions.

	**Transient vertigo (*n* = 16)**	**Continuous vertigo (*n* = 23)**	***P***
ACI	5 (31.3)	6 (26.1)	0.35
PCI	11 (68.8)	17 (73.9)	0.29
PICA	6 (37.5)	5 (21.7)	0.04
BA	4 (25.0)	10 (43.5)	0.01
PCA	1 (6.3)	1 (4.3)	0.67
BA+PCA	0	1 (4.3)

*ACI, anterior circulation infarction; PCI, posterior circulation infarction; PICA, posterior inferior cerebellar artery; BA, basilar artery; PCA, posterior cerebral artery*.

#### Vascular risk factors and clinical characteristics for stroke and hypoperfusion

Stroke was more frequent among patients with history of stroke, hypertension, hyperhomocysteinemia, diabetes mellitus, or cerebrovascular stenosis, compared to patients without cerebrovascular risk factors. There were significant differences between the stroke group and non-stroke group in the incidence of continuous vertigo and rotatory nystagmus; however, the overall incidences of nystagmus and sympathetic symptoms were higher in the non-stroke cohort than in the stroke cohort (Table 2).

**Table 2:** Cerebrovascular risk factors and clinical characteristics stroke and hypoperfusion.

	**DWI**		***p***	**PWI (non-stroke)**		***P***
	**Stroke (*n* = 39)**	**Non-stroke (*n* = 118)**		**Hypoperfusion (*n* = 68)**	**Non-hypoperfusion (*n* = 50)**
Age (year)	62.6 ± 11.1	59.0 ± 12.9	0.12	60.7 ± 13.7	56.6 ± 11.6	0.09
Male gender (*n*, %)	23 (59.0)	55 (46.6)	0.18	28 (41.2)	27 (54.0)	0.18
Hypertension (*n*,%)	32 (82.1)	71 (60.1)	0.01	42 (61.8)	29 (58.0)	0.68
Diabetes mellitus (*n*, %)	23 (59.0)	36 (30.5)	0.00	24 (35.3)	12 (24.0)	0.19
Dyslipidemia (*n*, %)	21 (53.8)	82 (69.5)	0.08	45 (66.2)	37 (74.0)	0.36
Coronary heart disease (*n*, %)	6 (15.4)	28 (23.7)	0.27	17 (25.0)	11 (22.0)	0.71
Smoking (*n*, %)	14 (35.9)	27 (22.9)	0.11	11 (16.2)	16 (32.0)	0.04
Alcoholism (*n*, %)	10 (25.6)	18 (15.3)	0.14	10 (14.7)	8 (16.0)	0.85
Stroke history (*n*, %)	13 (33.3)	13 (11.0)	0.00	9 (13.2)	4 (8.0)	0.37
Hyperhomocysteinemia (*n*, %)	13 (33.3)	21 (17.8)	0.04	12 (17.6)	11 (22.0)	0.31
Hyperuricemia (*n*, %)	0 (0)	3 (2.5)	0.32	0 (0)	1 (2.0)	1.00
History of vertigo (*n*, %)	0 (0)	7 (5.9)	0.12	3 (4.4)	2 (4.0)	0.71
Gait instability (*n*, %)	24 (61.5)	64 (54.2)	0.43	1 (1.5)	24 (48.0)	0.00
Hearing impairment (*n*, %)	1 (2.6)	6 (5.1)	0.51	1 (1.5)	3 (6.0)	0.70
Trigger factors (*n*, %)	2 (5.1)	4 (3.4)	0.62	55 (80.9)	3 (6.0)	0.41
Nystagmus (*n*, %)	23 (59.0)	89 (75.4)	0.04	16 (23.5)	34 (68.0)	0.00
Continuous vertigo (*n*, %)	23 (59.0)	41 (34.7)	0.01	21 (30.9)	20 (40.0)	0.30
Sympathetic symptoms (*n*, %)	29 (74.4)	106 (89.8)	0.02	63 (92.6)	43 (86.0)	0.24

*DWI, diffusionweighted imaging; PWI, perfusionweighted imaging*.

There were significant differences between patients with hypoperfusion and patients without non-hypoperfusion who had a history of smoking, nystagmus, and gait instability (Table 2).

#### Radiological imaging risk factors for stroke and hypoperfusion

Stroke and non-stroke groups differed significantly in terms of mean diameter of the basilar artery (*P* = 0.02) and VA stenosis or hypoplasia (*P* = 0.00). PWI maps revealed significant differences between stroke and non-stroke groups in terms of rrCBV of pons, rTTP of centrum semiovale, rMTT, and rTTP of cerebellum ROIs (*P* = 0.01, 0.01, 0.01, 0.00, respectively; Table 3). However, the curvature index of left vertebral artery (*P* = 0.01) and levels of VA stenosis or hypoplasia (*P* = 0.00) were similar in the hypoperfusion and non-hypoperfusion groups. As for the PWI parameters, only rTTP of cerebellum ROIs was found to differ significantly between hypoperfusion and non-hypoperfusion groups (*P* = 0.00; Table 3).

**Table 3:** Risk factors for stroke and hypoperfusion on radiological imaging.

	**DWI**		***p***	**PWI (non-stroke)**		***P***
	**Stroke (*n* = 39)**	**Non-stroke (*n* = 118)**		**Hypoperfusion (*n* = 68)**	**Non-hypoperfusion (*n* = 50)**
Diameters of RVA	2.76 ± 0.68	2.80 ± 0.66	0.77	2.76 ± 0.68	2.85 ± 0.62	0.44
Diameters of LVA	3.19 ± 0.67	3.22 ± 0.66	0.83	3.28 ± 0.58	3.13 ± 0.75	0.22
Actual lengths of RVA	114.23 ± 12.45	113.87 ± 9.06	0.85	114.08 ± 9.75	106.37 ± 7.38	0.78
Actual lengths of LVA	118.18 ± 13.66	118.62 ± 11.28	0.84	119.33 ± 11.45	117.64 ± 11.09	0.42
Straight lengths of RVA	105.44 ± 9.52	105.71 ± 7.89	0.86	105.23 ± 8.27	106.37 ± 7.38	0.44
Straight lengths of LVA	107.08 ± 15.13	106.18 ± 11.48	0.70	105.09 ± 11.58	107.66 ± 11.29	0.23
Curvature index of RVA	8.36 ± 6.98	7.80 ± 5.33	0.69	8.50 ± 5.95	6.85 ± 4.21	0.10
Curvature index of LVA	10.90 ± 7.44	11.84 ± 8.60	0.72	13.52 ± 9.37	9.56 ± 6.86	0.01
Diameters of BA	3.12 ± 1.54	3.14 ± 0.62	0.02	3.18 ± 0.63	3.09 ± 0.62	0.44
Actual length of BA	26.07 ± 5.83	27.32 ± 6.81	0.09	26.73 ± 5.25	28.13 ± 8.49	0.57
Straight length of BA	24.45 ± 5.54	24.76 ± 5.17	0.75	23.97 ± 4.10	25.83 ± 6.23	0.05
Ratio of BA length	1.07 ± 0.50	1.10 ± 0.16	0.45	1.12 ± 0.14	1.06 ± 0.17	0.18
BA bending length	3.12 ± 3.00	3.67 ± 2.93	0.18	3.55 ± 2.99	3.83 ± 2.88	0.19
Angle degree of VAs	72.11 ± 38.97	67.17 ± 29.03	0.40	67.89 ± 27.93	66.18 ± 30.72	0.75
VA stenosis or hypoplasia	29 (74.4)	54 (45.8)	0.00	39 (57.4)	16 (32.0)	0.00
Fetal PCA	3(0.08)	2 (0.02)	0.10	2 (0.03)	0	0.51
rrCBF of medulla	6.79 ± 6.99	7.18 ± 7.56	0.87	6.47 ± 6.27	8.15 ± 9.00	0.23
rrCBV of medulla	0.61 ± 0.52	0.72 ± 0.76	0.99	0.64 ± 0.63	0.82 ± 0.90	0.38
rMTT of medulla	0.97 ± 1.05	0.90 ± 1.05	0.94	1.05 ± 1.22	0.68 ± 0.71	0.15
rTTP of medulla	0.77 ± 0.92	0.71 ± 0.69	0.99	0.78 ± 0.68	0.63 ± 0.71	0.25
rrCBF of cerebellum	6.90 ± 7.64	5.00 ± 4.54	0.33	5.25 ± 4.87	4.66 ± 4.08	0.49
rrCBV of cerebellum	0.79 ± 0.97	0.53 ± 0.59	0.11	0.58 ± 0.61	0.46 ± 0.57	0.07
rMTT of cerebellum	1.47 ± 2.07	0.88 ± 1.29	0.01	1.10 ± 1.57	0.58 ± 0.67	0.13
rTTP of cerebellum	1.45 ± 1.69	0.51 ± 0.48	0.00	0.63 ± 0.50	0.34 ± 0.38	0.00
rrCBF of pons	4.89 ± 3.75	5.30 ± 4.91	0.89	5.68 ± 5.26	4.77 ± 4.39	0.32
rrCBV of pons	0.58 ± 0.40	0.46 ± 0.48	0.01	0.49 ± 0.56	0.42 ± 0.34	0.84
rMTT of pons	0.84 ± 1.08	0.63 ± 0.91	0.18	0.69 ± 0.98	0.55 ± 0.79	0.63
rTTP of pons	1.05 ± 1.29	0.64 ± 0.64	0.14	0.68 ± 0.73	0.60 ± 0.49	0.86
rrCBF of thalamus	3.24 ± 2.49	3.76 ± 3.74	0.91	3.98 ± 4.26	3.48 ± 2.89	0.72
rrCBV of thalamus	0.33 ± 0.27	0.35 ± 0.35	0.71	0.38 ± 0.38	0.32 ± 0.30	0.39
rMTT of thalamus	1.24 ± 4.00	0.53 ± 0.67	0.19	0.60 ± 0.78	0.44 ± 0.47	0.24
rTTP of thalamus	0.80 ± 1.15	0.49 ± 0.45	0.22	0.48 ± 0.38	0.52 ± 0.53	0.62
rrCBF of centrum semiovale	3.09 ± 3.13	2.68 ± 2.50	0.76	2.81 ± 2.69	2.51 ± 2.24	0.53
rrCBV of centrum semiovale	0.26 ± 0.25	0.30 ± 0.29	0.57	0.34 ± 0.31	0.25 ± 0.25	0.08
rMTT of centrum semiovale	0.91 ± 0.88	0.78 ± 0.93	0.12	0.88 ± 0.99	0.64 ± 0.82	0.06
rTTP of centrum semiovale	1.13 ± 1.23	0.65 ± 0.76	0.01	0.73 ± 0.83	0.54 ± 0.64	0.11
rrCBF of occipital lobe	2.44 (4.13)	2.29 (3.25)	0.71	2.49 (3.63)	2.20 (2.87)	0.89
rrCBV of occipital lobe	0.23 (0.25)	0.26 (0.36)	0.71	0.25 (0.38)	0.27 (0.38)	0.45
rMTT of occipital lobe	0.57 (0.98)	0.50 (0.75)	0.42	0.50 (0.69)	0.50 (0.96)	0.94
rTTP of occipital lobe	0.59 (1.20)	0.51 (0.77)	0.18	0.49 (0.74)	0.63 (0.98)	0.13
rrCBF of temporal lobe	3.49 (3.76)	2.69 (4.31)	0.26	3.36 (4.76)	2.15 (2.95)	0.03
rrCBV of temporal lobe	0.32 (0.37)	0.31 (0.44)	0.80	0.39 (0.58)	0.23 (0.34)	0.02
rMTT of temporal lobe	0.76 (1.26)	0.73 (1.00)	0.40	0.82 (0.88)	0.66 (1.11)	0.20
rTTP of temporal lobe	0.76 (1.39)	0.80 (1.01)	0.95	0.82 (1.01)	0.77 (1.13)	0.81

*DWI, diffusion-weighted imaging; PWI, perfusion-weighted imaging; RVA, right vertebral artery; LVA, left vertebral artery; BA, basilar artery; PCA, posterior cerebral artery; rCBF, relative cerebral blood flow; rCBV, relative cerebral blood volume; rMTT, relative mean transit time; rTTP, relative time to peak*.

In the current study, 3.2% (5/157) of patients with isolated vertigo had a fetal posterior cerebral artery (2 bilateral, 2 right, 1 left). The prevalence of fetal posterior cerebral artery was similar in the stroke and non-stroke groups. rrCBF, rrCBV, rMTT, and rTTP values for occipital and temporal lobe ROIs were similar in patients with vs. without fetal posterior cerebral arteries.

The PWI maps revealed that rrCBF, rrCBV, rMTT, and rTTP values for the occipital and temporal lobe ROIs were similar in the stroke and non-stroke groups (Table 3). Similarly, the prevalence of fetal posterior cerebral artery was similar in the hypoperfusion and non-hypoperfusion groups. Relative rCBF, rrCBV, rMTT, and rTTP values for occipital lobe ROIs were similar in the hypoperfusion and non-hypoperfusion groups. Though rrCBF and rrCBV of temporal lobe ROIs differed significantly between the hypoperfusion and non-hypoperfusion groups, neither rMTT nor rTTP of temporal lobes ROIs was significantly delayed in the hypoperfusion group (Table 3).

### Multivariate logistic regression analysis for stroke and hypoperfusion

Multivariate logistic regression indicated that diabetes mellitus (*P* = 0.049, 95%CI: 1.85–10.56, OR = 2.758), VA stenosis or hypoplasia (*P* = 0.023, 95%CI: 1.55–6.62, OR = 3.486), and rTTP of cerebellum (*P* = 0.002, 95%CI: 1.67–4.05, OR = 3.197) were independent risk factors for stroke (Table 4) and curvature index of LVA (*P* = 0.026, 95%CI: 1.00–1.10, OR = 2.049) and VA stenosis or hypoplasia (*P* = 0.009, 95%CI: 1.31–6.74, OR = 2.977) were independent risk factor for hypoperfusion (Table 5).

**Table 4:** Multivariate logistic regression analysis for stroke in patients with isolated vertigo (*n* = 157).

**Variants**	**B**	**S.E**,	**Wald**	**Sig.(*p*)**	**OR (95% C.I.)**
Hypertension	0.770	0.581	1.760	0.185	2.161 (1.01–7.57)
Diabetes mellitus	1.015	0.515	3.884	0.049	2.758 (1.85–10.56)
Stroke history	1.096	0.643	2.904	0.088	2.993 (0.57–4.78)
Hyperhomocysteinemia	0.499	0.544	0.843	0.358	1.647 (0.15–1.34)
Nystagmus	−0.804	0.561	2.054	0.152	0.447 (0.28–2.28)
Continuous vertigo	−0.219	0.533	0.169	0.681	0.803 (0.47–6.33)
Sympathetic symptoms	0.539	0.666	0.656	0.418	1.715 (0.76–1.86)
Mean BA diameter	0.170	0.230	0.547	0.460	1.186 (1.19–10.20)
VA stenosis or hypoplasia	1.249	0.548	5.195	0.023	3.486 (1.55–6.62)
rTTP of cerebellum	1.162	0.371	9.820	0.002	3.197 (1.67–4.05)
rCBV of pons	0.495	0.461	1.156	0.282	1.641 (1.19–10.20)
rTTP of centrum semiovale	0.325	0.257	1.603	0.205	1.384 (0.84–2.29)
Constant	−4.682	1.275	13.494	0.000

*VA, vertebral artery; rCBV, relative cerebral blood volume; rTTP, relative time to peak*.

**Table 5:** Multivariate logistic regression analysis for hypoperfusion in patients with isolated vertigo (*n* = 118).

**Variants**	**B**	**S.E**,	**Wals**	**Sig**.	**Exp (B)**
Smoking	−0.595	0.485	1.508	0.219	0.551 (0.21–1.43)
Curvature index of LVA	0.048	0.026	3.379	0.026	2.049 (1.00–1.10)
Line length of BA	−0.095	0.050	3.625	0.057	0.909 (0.83–1.00)
VA stenosis or hypoplasia	1.091	0.417	6.841	0.009	2.977 (1.31–6.74)
Constant	1.775	1.240	2.049	0.152

*LVA, left vertebral artery; BA, basilar artery*.

## Discussion

In this study of 157 patients with isolated vertigo accompanied by cerebrovascular risk factors, the prevalence of stroke was 24.8%. The prevalence of cerebellar hypoperfusion for which initial hypoperfusion was normalized on follow-up images was 41.2%. Among all patients with isolated vertigo without recent infarctions, hypoperfusion was found in 57.6%. More interestingly, diabetes mellitus, VA stenosis or hypoplasia and rTTP of cerebellum were independent risk factors for stroke and curvature index of LVA, VA stenosis or hypoplasia were independent risk factor for hypoperfusion. Accurate identification of vertigo/dizziness secondary to ischemia and risk factors may lead to appropriate timely intervention, minimizing stroke-related damage, and help in tailoring the most appropriate therapy for patients.

A previous study showed that the prevalence of stroke among patients with vertigo ranges from 3.2~25% (17–19). One recent study discovered that stroke was diagnosed in 27% of patients with acute transient vestibular syndrome (1). The present study used 3.0T MRI and recruited patients with isolated vertigo with cerebrovascular risk factor load may contribute to the higher detection rate of small infarct lesions and hypoperfusion. Also, considering the high incidence of stroke/hypoperfusion in patients with isolated vertigo and it is of great importance to find predictors for stroke/hypoperfusion for these vertiginous patients in order to perform timely appropriate treatment (6).

Previous studies have shown that central isolated vertigo is caused by lesions restricted to vestibular nuclei, cerebellar flocculus, tonsil, nodulus, inferior cerebellar peduncle, or nucleus prepositus hypoglossi (3, 17). The present study interestingly found that the infarction patients with transient vertigo usually occurred in the cerebellum, while the infarction patients with persistent vertigo usually were in the brain-stem. A disequilibrium of perfusion in bilateral cerebellum implies that hypoperfusion in the posterior circulation may lead to recurrent transient isolated vertigo (9). Using a rat model of global hypoperfusion, the researchers found that the vestibular structures were vulnerable to ischemia more than any other structures in the brainstem and cerebellum, and the medial vestibular nucleus was the most vulnerable (20). These two findings confirmed our hypothesis, but future studies to confirm these findings should include a larger sample size than that achieved for this study.

Previous studies have found VAH may cause hypoperfusion in the area of the posterior inferior cerebellar artery (13, 15). Ahn et al. (21) describes two patients with recurrent isolated vertigo and subsequent cerebellar infarction, probably because of unilateral hypoplastic vertebral artery. In a double-blind retrospective cohort study, results show 85.7% of the patients complaining of isolated positional vertigo with at least three stroke risk factors, had a vertebral artery abnormality as stenotic or hypoplastic (22). A study revealed that the risk of vascular events or long-time risk of stroke was higher in patients with vestibular symptom associated with more vascular risk factors and silent infarctions in CT imaging (8). A 4-year follow-up study showed that patients with vertigo are at higher risk (3.01-fold) for stroke than the general population. Furthermore, vertigo patients with 3 risk factors had a 5.51-fold increase in risk for stroke, compared to patients with vertigo but without risk factors (23). Furthermore, diabetes mellitus can accelerate intracranial atherosclerosis, especially in the posterior cerebral circulation regions, with a tendency to invade the deep perforating branches of the basilar artery (24), which constitutes the main blood supply of the brainstem. In addition, the autonomic nervous enuresis of the posterior circulation is less extensive than for the anterior circulation, which also contributes to the increased risk of PCI in patients with diabetic mellitus (25).

Interestingly, LVA curvature index was associated with lower perfusion, and cerebellar rTTP was an independent risk factor for stroke. It has been widely accepted that TTP and MTT are sensitive indices for hypoperfusion of the brainstem unrelated to CBF or CBV (9). To our knowledge, this is the first study to investigate the effects of VA curvature on hemodynamicsas captured by PWI. Additional studies will be necessary to confirm the findings presented above. Previous research from our laboratory showed that the combination of cerebrovascular risk factors and tortuosity of the vertebrobasilar artery may lead to vascular vertigo (26). One study showed that increased vertebral artery tortuosity was associated with earlier age at dissection and death in children and young adults with connective tissue disorders (16). The findings presented in this study demonstrate the importance of vertebrobasilar radiological evaluations to assess hypoplasia, tortuosity and PWI in patients complaining of isolated positional vertigo/dizziness of unexplained etiology who also present with increased risk for stroke. The findings presented above also show that presence of a fetal posterior cerebral artery was not a significant risk factor for stroke and/or hypoperfusion in the posterior circulation. This effect may be related to the limited number of patients with fetal posterior cerebral arteries included in the study.

This study had several limitations. First, it was a prospective, cross-sectional study that was performed at a single center. A relatively small sample may have contributed to selection bias. Second, the proportion of stroke correlated with isolated vertigo may have been underestimated. The study population did not include patients with history of mild stroke, who were often unwilling to visit the hospital when symptoms had spontaneously resolved. Third, brain MRI may have yielded false-negative results in patients with small infarction lesions; some patients classified as “non-stroke” may actually have had strokes that passed without notice. Fourth, no systematic evaluation was undertaken to assess degree of vertigo or to identify factors that may exacerbate or alleviate symptoms, truncal ataxia, or headache. These important clinical features were therefore not compared between groups. Finally, we were not able to exclude patients with conditions such as basilar migraine, which is also associated with deficits in perfusion.

## Conclusion

In summary, clinicians should consider the high prevalence of stroke and hypoperfusion in patients with isolated vertigo and vascular risk factors. Some patients presenting with isolated vertigo without lesions in DWI may even have changes in cerebral hemodynamics that can be detected by PWI. Such changes in cerebral hemodynamics may be associated with certain risk factors (e.g., VA stenosis or hypoplasia, VA curvature index). In the future, the link between hypoperfusion and VA hypoplasia or curvature index should be further studied in order to elucidate the etiology of isolated vertigo.

## Author contributions

DPZ and HRL designed and conceptualized study, analyzed the data, drafted the manuscript for intellectual content. MZ interpreted the data, revised the manuscript for intellectual content. QKM, SY, YFP, HLZ, and SLZ were major role in the acquisition of data.

### Conflict of interest statement

The authors declare that the research was conducted in the absence of any commercial or financial relationships that could be construed as a potential conflict of interest.
