# Erratum: Prevalence of Stroke and Hypoperfusion in Patients With Isolated Vertigo and Vascular Risk Factors

**DOI:** 10.3389/fneur.2018.01110

**Published:** 2018-12-13

**Authors:** Frontiers Production Office

**Affiliations:** Frontiers Production Office, Frontiers, Lausanne, Switzerland

**Keywords:** vertigo, stroke, hypoperfusion, vertebral artery, basilar artery, vessel curvature

Reason for Erratum:

In the published article, there was an error in affiliation 1. Instead of “Department of Neurology, Henan University of Chinese Medicine, Zhengzhou, China,” it should be “Department of Neurology, The First Affiliated Hospital of Henan University of Chinese Medicine, Zhengzhou, China.”

The publisher apologizes for this error and states that this does not change the scientific conclusions of the article in any way. The original article has been updated.

